# Publisher’s note

**DOI:** 10.1080/26410397.2020.1843239

**Published:** 2020-11-17

**Article title:** The integration of sexual and reproductive health and rights into universal health coverage: a FIGO perspective

**Authors:** Faysal El Kak

**Journal:** Sexual and Reproductive Health Matters

**Bibliometrics:** Volume 28, Number 1, article 1829796

**DOI:** https://doi.org/10.1080/26410397.2020.1829796

This article was originally published in issue 28.1. The article belongs instead to issue 28.2, a themed issue on “Universal Health Coverage: Sexual and Reproductive Rights in Focus.”

The article has now been republished in the correct issue, 28.2.

